# Fluid balance and renal replacement therapy initiation strategy: a secondary analysis of the STARRT-AKI trial

**DOI:** 10.1186/s13054-022-04229-0

**Published:** 2022-11-24

**Authors:** Ron Wald, Brian Kirkham, Bruno R. daCosta, Ehsan Ghamarian, Neill K. J. Adhikari, William Beaubien-Souligny, Rinaldo Bellomo, Martin P. Gallagher, Stuart Goldstein, Eric A. J. Hoste, Kathleen D. Liu, Javier A. Neyra, Marlies Ostermann, Paul M. Palevsky, Antoine Schneider, Suvi T. Vaara, Sean M. Bagshaw

**Affiliations:** 1 Division of Nephrology, St. Michael’s Hospital, University of Toronto, 61 Queen Street East, 9-140, Toronto, ON M5C 2T2 Canada; 2 Li Ka Shing Knowledge Institute of St. Michael’s Hospital, Toronto, ON Canada; 3 Applied Health Research Centre, St. Michael’s Hospital, Toronto, ON Canada; 4 Department of Critical Care Medicine, Sunnybrook Health Sciences Centre and Interdepartmental Division of Critical Care Medicine, University of Toronto, Toronto, Canada; 5 Division of Nephrology, Centre Hospitalier de l’Université de Montréal, Montréal, QC Canada; 6 Department of Critical Care, School of Medicine, The University of Melbourne, Parkville, Melbourne, VIC Australia; 7 Australian and New Zealand Intensive Care Research Centre, School of Public Health and Preventive Medicine, Monash University, Melbourne, Australia; 8 Department of Intensive Care, Austin Hospital, Melbourne, Australia; 9 Department of Intensive Care, Royal Melbourne Hospital, Melbourne, Australia; 10 The George Institute for Global Health, UNSW, Sydney, Australia; 11 Division of Nephrology, Cincinnati Children’s Hospital, Cincinnati, OH USA; 12 Intensive Care Unit, Department of Internal Medicine and Pediatrics, Ghent University Hospital, Ghent University, Ghent, Belgium; 13 Research Foundation-Flanders (FWO), Brussels, Belgium; 14 Departments of Medicine and Anesthesia, University of California, San Francisco, CA USA; 15 Division of Nephrology, University of Alabama Birmingham, Birmingham, AB USA; 16 Department of Critical Care, King’s College London, Guy’s & St Thomas’ Hospital, London, UK; 17 VA Pittsburgh Healthcare System and University of Pittsburgh, Pittsburgh, PA USA; 18 Adult Intensive Care Unit, Centre Hospitalier Universitaire Vaudois (CHUV), Lausanne, Switzerland; 19 Division of Intensive Care Medicine, Department of Anesthesiology, Intensive Care and Pain Medicine, University of Helsinki and Helsinki University Hospital, Helsinki, Finland; 20 Department of Critical Care Medicine, Faculty of Medicine and Dentistry, University of Alberta and Alberta Health Services, Edmonton, AB Canada

**Keywords:** Acute kidney injury, Renal replacement therapy, Randomized controlled trial, Fluid balance, Clinical outcomes

## Abstract

**Background:**

Among critically ill patients with acute kidney injury (AKI), earlier initiation of renal replacement therapy (RRT) may mitigate fluid accumulation and confer better outcomes among individuals with greater fluid overload at randomization.

**Methods:**

We conducted a pre-planned post hoc analysis of the STandard versus Accelerated initiation of Renal Replacement Therapy in Acute Kidney Injury (STARRT-AKI) trial. We evaluated the effect of accelerated RRT initiation on cumulative fluid balance over the course of 14 days following randomization using mixed models after censoring for death and ICU discharge. We assessed the modifying effect of baseline fluid balance on the impact of RRT initiation strategy on key clinical outcomes. Patients were categorized in quartiles of baseline fluid balance, and the effect of accelerated versus standard RRT initiation on clinical outcomes was assessed in each quartile using risk ratios (95% CI) for categorical variables and mean differences (95% CI) for continuous variables.

**Results:**

Among 2927 patients in the modified intention-to-treat analysis, 2738 had available data on baseline fluid balance and 2716 (92.8%) had at least one day of fluid balance data following randomization. Over the subsequent 14 days, participants allocated to the accelerated strategy had a lower cumulative fluid balance compared to those in the standard strategy (4509 (− 728 to 11,698) versus 5646 (0 to 13,151) mL, *p* = 0.03). Accelerated RRT initiation did not confer greater 90-day survival in any of the baseline fluid balance quartiles (quartile 1: RR 1.11 (95% CI 0.92 to 1.34), quartile 2: RR 1.03 (0.87 to 1.21); quartile 3: RR 1.08 (95% CI 0.91 to 1.27) and quartile 4: RR 0.87 (95% CI 0.73 to 1.03), *p* value for trend 0.08).

**Conclusions:**

Earlier RRT initiation in critically ill patients with AKI conferred a modest attenuation of cumulative fluid balance. Nonetheless, among patients with greater fluid accumulation at randomization, accelerated RRT initiation did not have an impact on all-cause mortality.

*Trial registration*: ClinicalTrials.gov number, https://clinicaltrials.gov/ct2/show/NCT02568722, registered October 6, 2015.

**Supplementary Information:**

The online version contains supplementary material available at 10.1186/s13054-022-04229-0.

## Background

The administration of fluid is a crucial part of resuscitation in critical illness but at some point, fluid administration may become deleterious to patients as pathologic fluid overload impairs the function of multiple organs [1]. Fluid accumulation is common in critically ill patients and exacerbated in the presence of acute kidney injury (AKI) [2–4]. Fluid accumulation may be mitigated by fluid-sparing resuscitation strategies that minimize nonessential fluid intake such as maintenance infusions, medications and nutrition [5]. In patients with excess fluid accumulation, diuretics can promote sodium and water excretion, but their efficacy may be limited in the setting of AKI. As a result, extracorporeal fluid removal is especially relevant in patients with severe AKI. There remains considerable debate about the optimal conditions for fluid removal and the rate at which this should be performed [6].

The STandard versus Accelerated initiation of Renal Replacement Therapy in Acute Kidney Injury (STARRT-AKI) trial was an international multicenter trial that compared two strategies of renal replacement therapy (RRT) initiation in patients with severe AKI who had no conventional indications for urgent RRT initiation. Patients were allocated to an accelerated strategy, which entailed RRT initiation shortly after meeting trial eligibility or to a standard strategy of structured RRT deferral until an AKI-related emergency occurred or another requirement for RRT arose based on the judgement of the attending clinician [7]. In this planned secondary analysis of the STARRT-AKI trial, we tested the hypothesis that accelerated RRT initiation would attenuate fluid accumulation. Secondarily, we sought to determine whether fluid balance at baseline modified the relationship between the study treatment (RRT initiation strategy) and clinical outcomes. Specifically, we hypothesized that accelerated initiation of RRT, as compared to the standard strategy, would confer a lower risk of death among those with greater cumulative fluid balance at baseline.

## Methods

The STARRT-AKI trial enrolled 3019 critically ill adults with severe AKI (Stage 2–3 using the Kidney Disease Improving Global Outcomes classification) at 168 centers in 15 countries [7]. Key exclusions were an urgent reason for RRT initiation (serum potassium > 5.5 mmol/L, serum bicarbonate < 15 mmol/L), a concomitant intoxication requiring RRT, a clinical decision not to pursue RRT due to restrictions on the escalation of care, recent receipt of RRT and advanced chronic kidney disease. Finally, patients were excluded if their clinician(s) felt that urgent RRT was mandated or that deferral of RRT was mandated due to a perception of imminent kidney recovery. The trial protocol, statistical analysis plan and the details of the trial’s main findings have been previously published [7–9]. STARRT-AKI was registered at ClinicalTrials.gov (ClinicalTrials.gov Identifier: NCT02568722, registered October 6, 2015). Ethics approval was granted by research ethics boards at all participating sites, and consent to participate was provided by patients or substitute decision makers; deferred or waived consent was permitted in certain jurisdictions as per local policy.

This planned secondary analysis aimed to evaluate the effect of an accelerated strategy of RRT initiation on fluid accumulation. First, among the 2927 patients who were eligible for the modified intention to treat analysis, we evaluated the difference in cumulative fluid balance, by randomized group, from the day of randomization through their entire ICU stay or up to day 14. We analyzed the intergroup differences in cumulative fluid balance within the following subgroups: male versus female, Simplified Acute Physiology Score (SAPS) II score > 58 versus ≤ 58, septic versus non-septic, estimated baseline glomerular filtration rate (eGFR) < 45 versus ≥ 45 mL/min/1.73 m2, medical versus surgical admission categories and geographic regions (North America, Australia-New Zealand, Europe, South America/Asia). As a sensitivity analysis, we repeated this assessment by considering only patients who received the RRT strategy to which they were allocated in addition to performing an as-treated analysis whereby patients who crossed over from their allocated arm to the opposite arm were analyzed based on the RRT initiation strategy that they actually received. In all cases, patients were censored at the time of death or ICU discharge.

We divided the trial’s intention to treat cohort into quartiles based on participants’ fluid balance at the time of randomization, which reflected the balance of all inputs and outputs from the time of ICU admission until randomization. We then evaluated whether baseline fluid balance modified the effect of RRT initiation strategy on all-cause 90-day mortality (the primary outcome of the trial) as well as selected secondary outcomes including RRT dependence in surviving patients, a composite outcome of death or RRT dependence at 90 days, and mortality in the ICU, in-hospital and at 28 days. Other outcomes of interest included ICU length of stay, hospital length of stay, ICU-free days, ventilator-free days, vasoactive-free days and hospitalization-free days. We further analyzed the modifying effect of baseline fluid balance on all-cause mortality in patients with and without sepsis and ICU admission categories (medical versus surgical). We conducted sensitivity analyses in which fluid balance at randomization was evaluated in three different ways: as deciles; as percentage fluid overload ((fluid balance at randomization defined as balance of inputs/outputs since ICU admission)/patient’s weight) × 100) dichotomized as > or ≤ 10%; and as a continuous variable.

### Statistical analyses

Cumulative fluid balance was calculated on a daily basis from the day of randomization (day 0) through Day 14. Patients who left the ICU prior to Day 14 or who died were censored at the time of death or discharge from the ICU. The between-group difference in fluid balance was evaluated using a linear mixed-effects model to account for repeated measures of fluid balance for each patient. Between-group differences in cumulative fluid balance on each patient’s last day in ICU were evaluated using *t* tests.

All trial participants who were eligible for the modified intention to treat analysis were divided into quartiles based on their fluid balance at the time of randomization. The demographic and clinical characteristics of the patients in each quartile were summarized, using means (standard deviations) or medians (interquartile ranges) for continuous variables and numbers (%) for categorical variables. To test for inter-quartile trend, linear regression models were used for continuous variables and general linear models (GLM) with binomial family and log link function for categorical variables. A logistic regression model was used to assess the interaction between fluid balance on a continuous scale and RRT strategy and the association with 90-day all-cause mortality.

We evaluated effect estimates for each of the outcomes of interest within each quartile: risk ratios (95% CI) for categorical outcomes were estimated using binomial general linear models with log-links, and *p* value for trends was estimated as the interaction between quartile and outcome in the models. Mean differences (95% CI) for continuous outcomes were evaluated with *t* tests, and *p* values for trend were estimated with a linear regression model. For subgroup analyses, *p* values for the interaction between the subgroup, randomization allocation and fluid balance quartile corresponded to a 3-way ANOVA model. *P* values < 0.05 were considered significant. All analyses were conducted using R version 3.6.2.

## Results

### The effect of accelerated RRT initiation on cumulative fluid balance

**Fig. 1:**
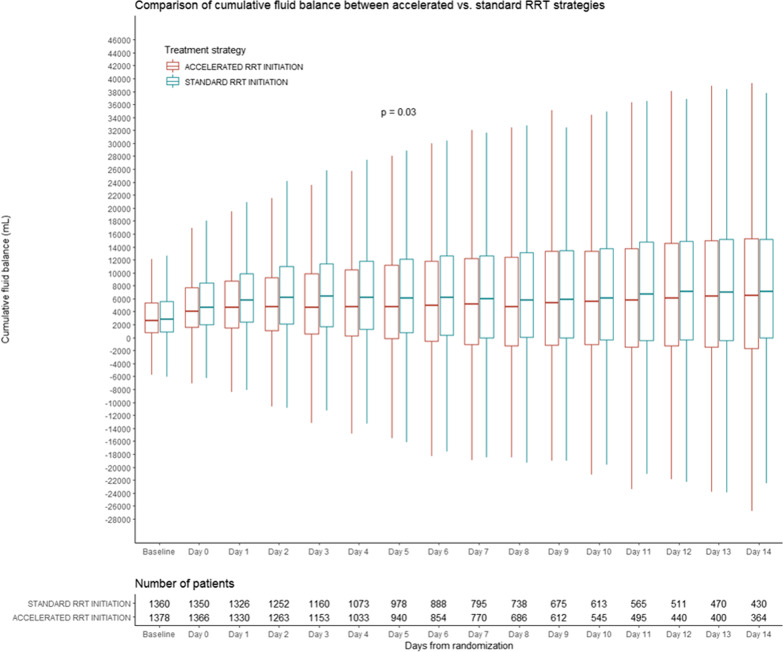
Cumulative fluid balance between days 0 and 14 comparing standard and accelerated RRT strategies

We evaluated 2738 patients who had fluid balance data available at the time of randomization. Of these, 2716 had at least one subsequent day of available fluid balance data. At the time of randomization, fluid accumulation was similar in patients allocated to both treatment strategies (accelerated 2581 (IQR 820–5362) versus standard 2819 (IQR 836–5603) mL). Over the subsequent 14 days, patients allocated to the accelerated strategy had a lower median cumulative fluid balance compared to patients allocated to the standard strategy (4509 (− 728 to 11,698) versus 5646 (0 to 13,151) mL, *p* = 0.03) (Fig. 1). Results were similar in sensitivity analyses that focused on the per-protocol and as-treated populations (Table 1). Among the 913 patients who were alive and still in the ICU at 14 days following randomization, fluid balance was not significantly lower among those allocated to the accelerated strategy compared to those allocated to the standard strategy (8071 (2768 to 16,589) versus 9979 (4054 to 19,261) mL, *p* = 0.1).

**Table 1:** Median cumulative fluid balance through day 14

	Accelerated, mL	Standard, mL	*p* value (mixed model)
Modified intent to treat	*N* = 1366	*N* = 1350	
Fluid accumulation days 0–14, censored at death or ICU discharge	4509 (− 728, 11,698)	5646 (0, 13,151)	0.03
Per-protocol	*N* = 1266	*N* = 1290	
Fluid accumulation days 0–14, censored at death or ICU discharge	4474 (− 743, 11,985)	5602 (2, 13,080)	0.05
As-treated	*N* = 1326	*N* = 1390	
Fluid accumulation days 0–14, censored at death or ICU discharge	4554 (− 733, 12,010)	5546 (0, 12,785)	0.10
mITT limited to ICU survivors at day 14	*N* = 450	*N* = 463	
Fluid accumulation days 0–14	8071 (2768, 16,589)	9979 (4054, 19,261)	0.1

All values are expressed as medians (interquartile range)

Modified intention to treat (mITT) population includes those who were analyzed in the arm to which they were allocated and comprises 2716 participants who had baseline data on fluid balance in addition to at least one more day of fluid balance data

The per-protocol analysis was limited to individuals for whom the RRT initiation strategy reflected the arm to which they were randomized (i.e., commenced RRT within 12 h of full eligibility in the accelerated arm; did not commence RRT within 12 h of meeting full eligibility in the standard arm)

The as-treated analysis included all patients and analyzed based on the RRT initiation strategy that they actually received

Across subgroups, fluid accumulation was generally attenuated among patients allocated to the accelerated strategy (Additional file 1: Table S1). There were no significant interactions between the allocated RRT strategy and any pre-specified subgroup for the outcome of fluid accumulation.

### Characterization of patients across quartiles of baseline fluid balance

Trial participants were categorized by ascending quartiles of fluid balance at randomization (Table 2). The median (interquartile range) baseline cumulative fluid balance differed substantially across quartiles: quartile 1: 125 (− 272, 487) mL; quartile 2: 1700 (1281, 2150) mL; quartile 3: 3907 (3250, 4625) mL and quartile 4: 9084 (6837, 12,279) mL. Patients with greater baseline fluid balance were more likely to have a surgical indication for ICU admission, a diagnosis of sepsis, a higher acuity of illness, defined by SAPS II and Sequential Organ Failure Assessment (SOFA) scores, were more likely to be receiving mechanical ventilation and vasoactive medications at randomization, and less likely to have received diuretic therapy. Baseline fluid balance was also associated with significant differences in acute physiology and laboratory parameters (Table 2).

**Table 2:** Patient characteristics at randomization by quartiles of baseline fluid balance

	Quartile 1 N = 685	Quartile 2 N = 685	Quartile 3 N = 684	Quartile 4 N = 684	*p* value for trend
Cumulative fluid balance, mL	125 (− 272, 487)	1700 (1281, 2150)	3907 (3250, 4625)	9084 (6837, 12,279)	< 0.01
Percent fluid overload, %	0 (0–1)	2 (2–3)	5 (4–6)	11 (8–15)	< 0.01
Age, years	66 (56–74)	67 (57–75)	67 (57–75)	65 (55–73)	0.24
Weight, kg	84.0 (70.0–100.50)	80.25 (70.0–98.0)	84.0 (70.0–98.8)	85.0 (72.0–101.0)	0.01
Baseline serum creatinine^a^, mg/dl	1.12 (0.84–1.59)	1.11 (0.83–1.56)	1.10 (0.83–1.53)	1.00 (0.76–1.32)	< 0.01
Baseline eGFR^b^, mL/min/1.73m^2^	62 (41–89)	64 (43–87)	64 (41–89)	75 (51–95)	< 0.01
Clinical Frailty Scale score	3 (1–4)	3 (1–4)	3 (1–4)	3 (1–4)	0.15
*Preexisting conditions*					
Hypertension	380 (56)	390 (57)	371 (54)	380 (56)	0.75
Diabetes mellitus	223 (33)	221 (32)	184 (27)	207 (30)	0.12
Heart failure	112 (16)	108 (16)	80 (12)	75 (11)	< 0.01
Coronary artery disease	176 (26)	163 (24)	131 (19)	136 (20)	< 0.01
Liver disease	86 (13)	80 (12)	71 (10)	86 (13)	0.81
Metastatic cancer	32 ( 5)	36 ( 5)	42 ( 6)	41 (6)	0.21
Hematologic malignancy	42 ( 6)	43 ( 6)	40 ( 6)	35 (5)	0.39
HIV/AIDS	5 ( 1)	7 ( 1)	6 ( 1)	6 (1)	0.85
*Admission category*					
Scheduled surgery	87 (13)	89 (13)	100 (15)	98 (14)	< 0.01
Unscheduled surgery	119 (17)	118 (17)	157 (23)	149 (22)	
Medical	479 (70)	478 (70)	427 (62)	437 (64)	
*AKI risk factors*					
Cardiopulmonary bypass	44 (6)	53 (8)	71 (10)	55 (8)	0.11
Aortic aneurysm repair	26 (4)	25 (4)	46 (7)	44 (6)	0.66
Major trauma	16 (2)	25 (4)	27 (4)	44 (6)	< 0.01
Radiocontrast exposure	151 (22)	175 (26)	187 (27)	206 (30)	0.99
Receipt of an aminoglycoside	65 ( 9)	68 (10)	81 (12)	68 (10)	0.16
Sepsis	333 (49)	417 (61)	416 (61)	420 (61)	< 0.01
Septic shock	215 (31)	322 (47)	338 (49)	331 (48)	< 0.01
SAPS II score	52 (42–65)	59 (46–72)	60 (49–74)	62 (50–74.3)	< 0.01
SOFA score	11 (8–13)	12 (9–14)	12 (10–14)	13 (11–15)	< 0.01
Mechanical ventilation	472 (69)	513 (75)	551 (81)	608 (89)	< 0.01
Vasoactive support	405 (59)	492 (72)	536 (78)	507 (74)	< 0.01
Diuretic therapy in preceding 24 h	293 (43)	241 (35)	209 (31)	213 (31)	< 0.01
Enteral nutrition	212 (31)	243 (35)	248 (36)	339 (50)	< 0.01
Total parenteral nutrition	83 (12)	84 (12)	79 (12)	89 (13)	0.72
*Physiological parameters*					
Heart rate, beats/min	105 (89–123)	105 (90–122.75)	110 (92.50–125)	109 (91–125)	0.01
Systolic blood pressure, mmHg	98 (85–120)	94 (83–112)	92 (81–107)	95 (84–110)	< 0.01
Temperature, °C	37.3 (36.6–38.1)	37.4 (36.6–38.1)	37.5 (36.8–38.3)	37.6 (36.9–38.3)	< 0.01
Glasgow Coma Scale	12 (5–15)	10 (3–15)	8 (3–14)	6 (3–11)	< 0.01
Urine output, mL/24 h	551 (178–1300)	500 (200–925)	399 (170–790)	500 (224–927)	< 0.01
*Laboratory parameters*					
Hemoglobin, g/dL	9.7 (8.3–11.6)	9.9 (8.4–11.7)	9.7 (8.3–11.4)	9.2 (8.0–10.7)	< 0.01
*White blood cell count—cells*					
× 10^9^/L	14 (9–20)	15 (10–21)	15 (10–22)	15 (10–21)	0.51
Platelets, cells × 10^9^/L	158 (90–234)	155 (88–231)	145 (87–216)	127 (73–214)	< 0.01
Serum bilirubin, mg/dL	0.88 (0.41–1.72)	0.88 (0.47–1.81)	0.88 (0.47–1.87)	0.99 (0.47–2.63)	0.74
Arterial pH	7.35 (7.28–7.40)	7.33 (7.27–7.39)	7.33 (7.25–7.38)	7.32 (7.26–7.39)	< 0.01
Serum bicarbonate, mmol/L	20 (17–23)	19 (16–22)	19 (17–22)	19 (16–22)	< 0.01
*Outcomes*					
90-day all-cause mortality	274 (40.0)	312 (45.5)	309 (45.2)	307 (44.9)	0.17
RRT dependence at 90 days	42 (10.2)	28 (7.6)	28 (7.5)	29 (7.8)	0.26
Ventilator-free days through day 28	18 (0–26)	12 (0–24)	12 (0–23)	6 (0–21)	< 0.01
Hospitalization-free days through day 90	34 (0–69)	7 (0–66)	0 (0–62)	0 (0–57)	< 0.01

All values expressed as medians with interquartile range or numbers (%)

The baseline serum creatinine level was defined as the most recent outpatient level obtained during the year preceding the current hospitalization. If this value was not available, the lowest serum creatinine level obtained during the current hospitalization was used to establish the baseline

^‡^The estimated glomerular filtration rate was calculated with the use of the Chronic Kidney Disease Epidemiology collaboration equation, which incorporates the baseline serum creatinine level, age, sex, and black race

^¶^Results for the Simplified Acute Physiology Score (SAPS) II range from 0 to 163, with higher scores indicating more severe disease and a higher risk of death

^‖^Scores on the Sequential Organ Failure Assessment (SOFA) range from 0 to 24, with higher scores indicating more severe disease and a higher risk of death

### The effect of accelerated RRT initiation on clinical outcomes across quartiles of baseline fluid balance

**Fig. 2:**
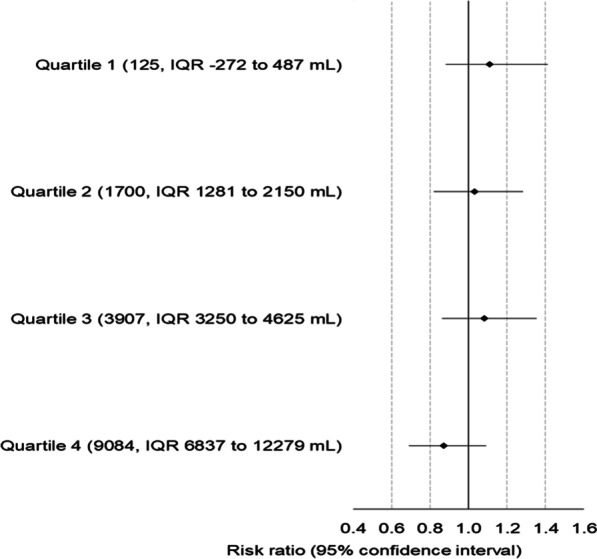
The effect of accelerated versus standard RRT initiation on 90-day mortality across quartiles of cumulative fluid balance at randomization

Accelerated initiation of RRT did not confer a reduction in 90-day mortality across any of the baseline fluid balance quartiles (quartile 1: RR 1.11 (95% CI 0.92 to 1.34); quartile 2: RR 1.03 (95% CI 0.87 to 1.21); quartile 3: RR 1.08 (95% CI 0.91 to 1.27) and quartile 4: RR 0.87 (95% CI 0.73 to 1.03), p-trend 0.08) compared to the standard RRT strategy (Table 3; Fig. 2). The difference in hospital-free days to day 90 favored the accelerated arm group as baseline fluid balance increased (*p* = 0.04 for trend). Patients in the highest baseline fluid balance quartile who were randomized to the accelerated arm had 4.6 (95% CI − 0.01 to 9.17) more hospital-free days. Fluid balance at the time of randomization did not modify the effect of accelerated RRT initiation on the other secondary outcomes (Table 3).

**Table 3:** The effect of accelerated RRT initiation on outcomes, by quartiles of baseline fluid balance

	Quartile 1			Quartile 2			Quartile 3			Quartile 4			*p* value for trend
	Acc *n* = 347	Std *n* = 338	RR (95% CI)	Acc *n* = 360	Std *n* = 325	RR (95% CI)	Acc *n* = 335	Std *n* = 349	RR (95% CI)	Acc n = 336	Std *n* = 348	RR (95% CI)	
*Outcome*													
90-day all-cause mortality, *n* (%)	146 (42.1)	128 (37.9)	1.11 (0.92 to 1.34)	166 (46.1)	146 (44.9)	1.03 (0.87 to 1.21)	157 (46.9)	152 (43.6)	1.08 (0.91 to 1.27)	140 (41.7)	167 (48.0)	0.87 (0.73 to 1.03)	0.08
RRT dependence at 90 days, *n* (%)	24 (12.0)	18 (8.6)	1.40 (0.79 to 2.54)	19 (9.8)	9 (5.1)	1.93 (0.92 to 4.37)	17 (9.6)	11 (5.7)	1.68 (0.82 to 3.59)	19 (9.9)	10 (5.6)	1.78 (0.87 to 3.89)	0.61
Death or RRT dependence at 90 days, *n* (%)	170 (49.0)	146 (43.2)	1.13 (0.96 to 1.34)	185 (51.4)	155 (47.7)	1.08 (0.93 to 1.26)	174 (51.9)	163 (46.7)	1.11 (0.96 to 1.30)	159 (47.3)	177 (50.9)	0.93 (0.80 to 1.08)	0.12
ICU mortality, *n* (%)	98 (28.2)	90 (26.6)	1.06 (0.83 to 1.36)	110 (30.6)	106 (32.6)	0.94 (0.75 to 1.17)	115 (34.3)	115 (33.0)	1.04 (0.84 to 1.29)	113 (33.7)	128 (36.8)	0.92 (0.75 to 1.12)	0.52
28-day mortality, *n* (%)	122 (35.2)	108 (32.0)	1.10 (0.89 to 1.36)	134 (37.2)	120 (36.9)	1.01 (0.83 to 1.23)	137 (40.9)	124 (35.5)	1.15 (0.95 to 1.40)	116 (34.5)	134 (38.5)	0.90 (0.73 to 1.09)	0.31
Hospital mortality, *n* (%)	121 (35.0)	106 (31.5)	1.11 (0.90 to 1.37)	140 (39.0)	125 (38.5)	1.01 (0.84 to 1.23)	137 (41.1)	128 (36.8)	1.12 (0.93 to 1.35)	125 (37.5)	151 (43.4)	0.87 (0.72 to 1.04)	0.14
			MD			MD			MD			MD	
ICU LOS, days (IQR)	7 (4, 13)	7 (4, 14)	− 0.1 (− 1.70 to 1.57)	8 (4,14)	8 (4,17)	− 1.6 (− 3.46 to 0.31)	8 (4,14)	10 (5, 17)	− 2.0 (− 3.84 to − 0.2)	10 (5, 18)	11 (6, 21)	− 1.8 (− 4.02 to 0.44)	0.19
Hospital LOS, days (IQR)	16 (8, 34)	18 (8, 32)	1.2 (− 2.72 to 5.03)	19 (8,36)	18 (7,36)	− 0.7 (− 4.35 to 2.97)	18 (8,35)	21 (11, 37)	− 2.4 (− 6.20 to 1.38)	23 (9, 43)	23 (9, 48)	− 1.0 (− 5.1 to 3.14)	0.35
Ventilator-free days@D28, days (IQR)	17 (0, 26)	19 (0, 26)	− 0.6 (− 2.43 to 1.21)	13 (0, 24)	10 (0, 24)	0.9 (− 0.87 to 2.63)	10 (0,24)	13 (0, 23)	− 0.1 (− 1.86 to 1.61)	8 (0, 21)	2 (0, 20)	1.4 (− 0.23 to 2.97)	0.21
Vasoactive-free days@D28, days IIQR)	22 (0, 26)	23 (0, 26)	− 0.5 (− 2.33 to 1.31)	20 (0, 26)	18 (0, 25)	0.9 (− 0.90 to 2.67)	20 (0,25)	19 (0, 25)	− 0.7 (− 2.51 to 1.09)	20 (0, 25)	18 (0, 25)	1.1 (− 0.63 to 2.91)	0.41
ICU-free days@D28, days (IQR)	12 (0, 22)	14 (0, 22)	− 0.5 (− 2.08 to 1.09)	8 (0, 20)	1 (0, 20)	0.9 (− 0.60 to 2.46)	1 (0,20)	5 (0, 19)	− 0.1 (− 1.64 to 1.42)	4 (0, 19)	0 (0, 16)	1.9 (0.43 to 3.28)	0.08
Hospitalization-free days@D90, days (IQR)	16 (0, 68)	39 (0, 69)	− 3.6 (− 8.71 to 1.58)	6 (0, 65)	8 (0, 67)	− 0.2 (− 5.13 to 4.68)	0 (0,62)	10 (0, 63)	− 1.3 (− 6.16 to 3.47)	10 (0, 59)	0 (0, 54)	4.6 (− 0.01 to 9.20)	0.04

*Acc* accelerated, *Std* standard, *ACM* all-cause mortality, *RRT* renal replacement therapy, *LOS* length of stay, *IQR* interquartile range, *MD* mean difference between (accelerated-standard)

### Subgroup analyses

The effect of accelerated RRT initiation on all-cause mortality was not modified by ascending quartiles of baseline fluid balance when sub-categorized by the presence or absence of sepsis or the category of ICU admission (Table 4).

**Table 4:** The effect of accelerated RRT initiation across quartiles of baseline fluid balance, further categorized by presence/absence of sepsis and principal reason for admission (surgical or medical)

	Quartile 1			Quartile 2			Quartile 3			Quartile 4			*p* value for trend	*p* value for interaction
	Acc	Std	RR (95% CI)	Acc	Std	RR (95% CI)	Acc	Std	RR (95% CI)	Acc	Std	RR (95% CI)		
*Septic patients, n* = *1586*														
90-day ACM, *n* (%)	70 (41.2)	64 (39.3)	1.05 (0.75 to 1.47)	121 (51.7)	92 (50.3)	1.03 (0.79 to 1.35)	95 (47.7)	98 (45.2)	1.06 (0.80 to 1.40)	85 (41.7)	119 (55.1)	0.76 (0.57 to 1.00)	0.13	0.21
*Non-septic patients, n* = *1152*														
90-day ACM, *n* (%)	76 (42.9)	64 (36.6)	1.17 (0.84 to 1.64)	45 (35.7)	54 (38.0)	0.94 (0.63 to 1.39)	62 (45.6)	54 (40.9)	1.11 (0.77 to 1.61)	55 (41.7)	48 (36.4)	1.15 (0.78 to 1.69)	0.98	
*Surgical patients, n* = *917*														
90-day ACM, *n* (%)	42 (37.8)	28 (29.5)	1.28 (0.8 to 2.09)	40 (36.7)	31 (31.6)	1.16 (0.73 to 1.87)	54 (41.2)	46 (36.5)	1.13 (0.76 to 1.68)	43 (34.4)	40 (32.8)	1.05 (0.68 to 1.62)	0.56	0.90
*Medical patients, n* = *1821*														
90-day ACM, *n* (%)	104 (44.1)	100 (41.2)	1.07 (0.81 to 1.41)	126 (50.2)	115 (50.7)	0.99 (0.77 to 1.28)	103 (50.5)	106 (47.5)	1.06 (0.81 to 1.39)	97 (46)	127 (56.2)	0.82 (0.63 to 1.06)	0.22	

*ACM* all-cause mortality, *Acc* accelerated, *Std* standard

### Sensitivity analyses

**Fig. 3:**
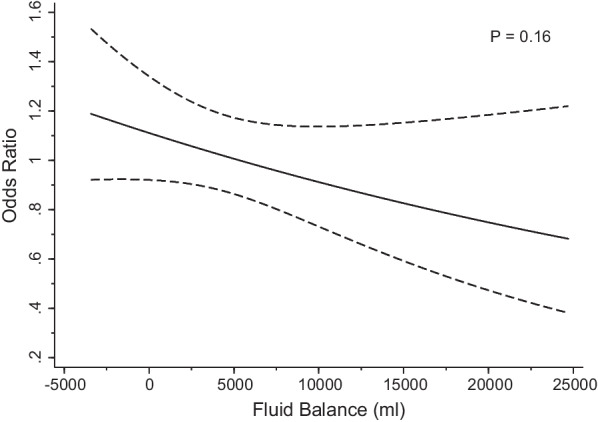
The effect of accelerated RRT initiation, as compared to standard RRT initiation, on all-cause mortality across the spectrum cumulative fluid balance at randomization

When fluid balance at randomization was categorized in deciles or dichotomized based on a percent fluid overload of ≤ or > 10%, accelerated RRT initiation had no effect on 90-day mortality or any of the secondary outcomes (Additional file 1: Tables S2 and S3). Similarly, accelerated RRT initiation had no significant effect on 90-day mortality across a spectrum of continuously measured levels of fluid balance (Fig. 3).

## Discussion

In a secondary analysis of a large multinational trial comparing accelerated versus standard RRT initiation in critically ill patients with AKI, we found that an accelerated strategy mediated a cumulative fluid balance that was lower by about 1 L during the 14 days that followed randomization. However, earlier RRT initiation did not confer improved survival among individuals with greater degrees of baseline fluid accumulation.

Fluid overload is a pervasive complication of AKI and has been associated with adverse outcomes [2, 4]. Extracorporeal ultrafiltration, provided in the context of RRT, can provide a reliable means of decongestion and achievement of net negative fluid balance. In patients with severe AKI but without otherwise pressing indications to commence RRT, mitigation of the adverse effects of positive fluid accumulation or maintenance of a relatively neutral fluid balance could conceivably justify earlier RRT initiation in the absence of other indications. In this regard, accelerated RRT initiation mediated a modestly lower net positive fluid balance compared to the standard strategy. This finding differs from the Initiation of Dialysis Early Versus Delayed in the Intensive Care Unit trial in which patients with Stage 3 AKI and septic shock were randomized to immediate RRT initiation versus a 48-h delay [10]. Despite a median delay to RRT initiation of 44 h in the delayed arm of the trial as compared to the early arm, fluid balance one week after randomization was no different between the early and delayed arms. We also found that earlier RRT initiation did not translate into a reduction in all-cause mortality or a meaningful improvement in other clinical outcomes across baseline fluid balance strata. Ventilator-free days, an outcome that could conceivably be impacted by more aggressive decongestion, was also not improved by accelerated RRT initiation even among patients with the greatest fluid balance at baseline [11].

There are several potential explanations for our findings. The STARRT-AKI intervention focused solely on the timing of RRT initiation and in the spirit of pragmatism did not protocolize any other aspect of the RRT prescription. As a result, the opportunity for more effective decongestion or mitigation of further accumulation via the earlier application of RRT in the accelerated arm may have been under-realized. This could have even led to inadequate ultrafiltration in patients with the greatest degrees of fluid overload, who theoretically could have benefited most from an accelerated initiation strategy. In addition, clinicians were permitted to use diuretics at any time at their discretion for patients in both arms of the trial and once RRT commenced, fluid removal targets were driven by clinician decision making. This issue is further compounded by the lack of clear criteria for what constitutes clinically relevant fluid overload in critically ill patients and uncertainty regarding optimal triggers and targets for extracorporeal fluid removal [12]. As a result, ultrafiltration was largely guided by clinician judgement, likely contributing to heterogeneity in practice.

Cumulative fluid balance since ICU admission was relatively modest (2500–3000 mL) at the time of randomization. This translates into a ~ 5% cumulative fluid balance indexed to baseline weight and is substantially lower than that observed in other cohorts of patients initiating acute RRT [13]. Greater sensitivity to the dangers of fluid overload in critically ill patients may have also led to the more modest fluid gains at the time of randomization. ICU practices that mitigate fluid accumulation include limiting fluid boluses and maintenance infusions, the concentration of infusions into smaller volumes and the more proactive use of diuretics; one or more of these practices may have moderated the degree of positive fluid balance that was observed in STARRT-AKI and could have minimized the effect of accelerated RRT initiation on overall fluid balance [3, 14]. In addition, prior to randomization, clinicians were asked to exclude individuals for whom immediate RRT was mandated. It is possible that some patients who were provisionally eligible for the trial were excluded by clinicians who felt that immediate RRT was necessary as a result of an unacceptable risk due to complications from preexisting fluid overload. In such patients, further deferral of RRT, as would be the case if the patient was randomized to the standard-strategy, might have been viewed as unethical [15].

This study has several strengths. Although there have been several trials comparing RRT initiation strategies in the setting of AKI in recent years [10, 16–18], only one other trial examined the effect of timing strategies on fluid balance [10], and there has never been an assessment of the modifying effect of baseline fluid balance on the relationship between RRT initiation strategy and clinical outcomes. Near-complete data on baseline fluid balance enabled a robust assessment of the effect of accelerated RRT initiation across different categories of fluid balance. Data were rigorously collected and reviewed using explicit criteria. The broad representation from patients around the world bolsters the generalizability of our findings.

This work has several limitations. Though we recorded data on fluid balance at trial randomization and follow-up, we did not collect information on factors that may have affected fluid balance, notably the nature of administered fluids and use of diuretics. While our findings suggest that an accelerated strategy of RRT initiation did not impact key clinical outcomes among individuals with the greatest degree of fluid accumulation, the pragmatic nature of the trial, whereby fluid management decisions were at the discretion of treating clinicians, could have moderated any effect of the accelerated RRT initiation strategy. Finally, fluid balance was characterized by the balance of inputs and outputs from the time the patient arrived in the ICU until randomization. Though these data are widely collected in critically ill patients, they may not necessarily be an accurate reflection of pathologic organ congestion. Importantly, fluid balance measures do not include insensible losses and often do not account for fluid intake or losses prior to ICU admission. In some cases a markedly positive fluid balance is the end-result of appropriate resuscitation. For example, a patient who presents to the ICU with profound hypovolemic shock and is aggressively resuscitated in order to restore hemodynamic stability will have an appropriately high fluid balance.

## Conclusions

In a recently completed international randomized trial, an accelerated strategy of RRT initiation conferred a modest reduction in cumulative fluid balance during the two weeks following enrollment. Fluid balance at the time of randomization was associated with several markers of illness severity. However, earlier initiation of RRT did not confer improved mortality but did lead to a greater number of hospital-free days in patients with the highest degree of fluid overload. Clinical trials that evaluate optimal ultrafiltration strategies in patients receiving RRT for AKI are urgently needed.

## Supplementary Information

**Additional file 1. Table S1**: Median fluid accumulation through Day 14, in the modified intention to treat population, stratified by subgroups. **Table S2**: The effect of accelerated vs standard RRT strategy on outcomes across deciles of baseline fluid balance. **Table S3**: The effect of accelerated vs standard RRT strategy on outcomes in patients with ≤ or > 10% fluid overload at baseline.

## Data Availability

The STARRT-AKI data sharing statement will generally align with the data sharing policy of The George Institute for Global Health (https://www.georgeinstitute.org/data-sharing-policy). The STARRT-AKI Co-Chairs and Steering Committee support the view that research data generated from publicly funded research should be made available for sharing to enhance public well-being, to maximize the potential knowledge gained, to reduce redundant research and to facilitate scientific discovery and innovation. Data sharing will be for the purposes of medical research and under the auspices of the consent under which the data were originally gathered. De-identified individual participant data collected during the STARRT-AKI trial will be shared beginning two years after the publication of the primary and secondary analyses, with no end date. Data will be made available to qualified researchers who provide a detailed and methodologically sound proposal with specific aims that are clearly outlined. To gain access, qualified researchers will need to sign a data sharing and access agreement and will need to confirm that data will only be used for the purpose for which data access was granted. Proposals should be directed to the trial Co-Chairs via email: bagshaw@ualberta.ca and Ron.Wald@unityhealth.to.
