# Supplementary material for: Fluid balance and renal replacement therapy initiation strategy: a secondary analysis of the STARRT-AKI trial
Source: Crit Care. 2022 Nov 24;26:360. doi: 10.1186/s13054-022-04229-0 (PMC9694606; doi:10.1186/s13054-022-04229-0)
Supplement: Supplementary file 1 — Additional file 1. Table S1: Median fluid accumulation through Day 14, in the modified intention to treat population, stratified by subgroups. Table S2: The effect of accelerated vs standard RRT strategy on outcomes across deciles of baseline fluid balance. Table S3: The effect of accelerated vs standard RRT strategy on outcomes in patients with ≤ or > 10% fluid overload at baseline. [file 13054_2022_4229_MOESM1_ESM.docx]

**Table S1. Median fluid accumulation through Day 14, in the modified intention to treat population, stratified by sub-groups**

|  | **Accelerated, mL** | **Standard, mL** | **p-value**  **(mixed model)** | **Interaction term** |
| --- | --- | --- | --- | --- |
| **Sex** |  |  |  |  |
| Male (n=1847) | 3944 (-1094, 11204) | 5664 (-65, 13360) | 0.02 | 0.26 |
| Female (n=869) | 5200 (192, 12025) | 5373 (356, 12588) | 0.73 |  |
| **SAPS** |  |  |  |  |
| >58 (n=1364) | 6086 (650 - 13325) | 7350 (1405, 15217) | 0.01 | 0.09 |
| <=58 (n=1352) | 3157 (-1559 - 9648) | 3668 (-1508, 10274) | 0.87 |  |
| **Sepsis** |  |  |  |  |
| Yes (n=620) | 5022 (-310 - 13741) | 7678 (2287, 15981) | 0.06 | 0.20 |
| No (n=2096) | 4302 (-732 - 11062) | 4908 (-406, 12080) | 0.13 |  |
| **ICU admission category** |  |  |  |  |
| Medical (n=1802) | 4518 (-826 - 12251) | 6119 (317, 14691) | 0.08 | 0.81 |
| Surgical (n=914) | 4448 (-407 - 10784) | 4736 (-522, 10986) | 0.22 |  |
| **Baseline eGFR** |  |  |  |  |
| >=45 (n=2021) | 4898 (-763 - 12126) | 5902 (338, 13639) | 0.04 | 0.84 |
| < 45 (n=695) | 3532 (-439 - 9794) | 4600 (-597, 12125) | 0.39 |  |
| **World Region** |  |  |  |  |
| North America (n=943) | 7122 (1268, 15910) | 8303 (1753, 16905) | 0.02 | 0.14 |
| AUNZ (n=533) | 1819 (-2168, 6616) | 2432 (-1101, 6917) | 0.77 |  |
| Europe (n=1031) | 5222 (14, 11656) | 6386 (605, 14374) | 0.58 |  |
| South America/Asia (n=209) | 64 (-3264, 4118) | 1290 (-2949, 6688) | 0.97 |  |
| **Age category** |  |  |  |  |
| > 65 (n=1424) | 4875 (-11, 11268) | 5745 (614, 12962) | 0.05 | 0.82 |
| <= 65 (n=1292) | 3881 (-1400, 11950) | 5400 (-584, 13371) | 0.23 |  |

All values expressed as medians with interquartile range.

Modified intention to treat (mITT) population includes those who were analyzed in the arm to which they were allocated and comprises 2716 participants who had baseline data on fluid balance in addition to at least one more day of fluid balance data

SAPS=Simplified Acute Physiology Score

eGFR=estimated glomerular filtration rate

**Table S2. The effect of accelerated vs standard RRT strategy on outcomes within deciles of baseline fluid balance**

| **Outcome** | **Decile 1** | **Decile 2** | **Decile 3** | **Decile 4** | **Decile 5** | **Decile 6** | **Decile 7** | **Decile 8** | **Decile 9** | **Decile 10** | **p-trend** |
| --- | --- | --- | --- | --- | --- | --- | --- | --- | --- | --- | --- |
|  | **RR (95% CI)** | **RR (95% CI)** | **RR (95% CI)** | **RR (95% CI)** | **RR (95% CI)** | **RR (95% CI)** | **RR (95% CI)** | **RR (95% CI)** | **RR (95% CI)** | **RR (95% CI)** |  |
| **90- day ACM, n(%)** | 1.13 (0.77 to 1.66) | 1.16 (0.8 to 1.67) | 1.03 (0.72 to 1.47) | 1.47 (1.02 to 2.13) | 0.72 (0.51 to 1.03) | 0.93 (0.65 to 1.33) | 1.08 (0.76 to 1.54) | 0.98 (0.69 to 1.38) | 0.97 (0.68 to 1.39) | 0.88 (0.62 to 1.25) | 0.20 |
| **RRT dependence at 90 days,** **n (%)** | 1.88 (0.71 to 5.45) | 1.33 (0.48 to 3.79) | 1.31 (0.51 to 3.55) | 2.17 (0.72 to 7.17) | 1.65 (0.36 to 11.51) | 1.71 (0.59 to 5.56) | 0.50 (0.07 to 2.31) | 3.88 (1.25 to 16.91) | 1.14 (0.27 to 4.83) | 1.72 (0.54 to 6.45) | 0.79 |
| **Death or RRT, n(%)** | 1.19 (0.83 to 1.71) | 1.16 (0.82 to 1.64) | 1.05 (0.76 to 1.48) | 1.48 (1.05 to 2.11) | 0.76 (0.54 to 1.08) | 1.00 (0.71 to 1.41) | 1.04 (0.74 to 1.46) | 1.11 (0.80 to 1.55) | 0.98 (0.69 to 1.39) | 0.94 (0.67 to 1.31) | 0.24 |
| **ICU mortality, n(%)** | 0.90 (0.55 to 1.47) | 1.11 (0.72 to 1.72) | 1.07 (0.69 to 1.67) | 1.22 (0.8 to 1.88) | 0.74 (0.49 to 1.13) | 1.01 (0.67 to 1.52) | 0.98 (0.65 to 1.49) | 0.86 (0.57 to 1.29) | 0.99 (0.66 to 1.48) | 1.04 (0.70 to 1.54) | 0.80 |
| **28-day mortality, n(%)** | 1.00 (0.64 to 1.56) | 1.17 (0.79 to 1.72) | 1.11 (0.75 to 1.67) | 1.37 (0.92 to 2.04) | 0.73 (0.50 to 1.08) | 1.05 (0.71 to 1.55) | 1.07 (0.73 to 1.57) | 0.97 (0.66 to 1.42) | 1.04 (0.69 to 1.54) | 0.97 (0.66 to 1.43) | 0.55 |
| **Hospital mortality, n(%)** | 1.07 (0.69 to 1.67) | 1.14 (0.77 to 1.70) | 1.09 (0.73 to 1.62) | 1.42 (0.96 to 2.10) | 0.73 (0.50 to 1.06) | 1.00 (0.68 to 1.47) | 1.10 (0.75 to 1.6) | 1.00 (0.68 to 1.46) | 0.90 (0.61 to 1.31) | 0.92 (0.64 to 1.33) | 0.29 |
|  | **MD (95% CI)** | **MD (95% CI)** | **MD (95% CI)** | **MD (95% CI)** | **MD (95% CI)** | **MD (95% CI)** | **MD (95% CI)** | **MD (95% CI)** | **MD (95% CI)** | **MD (95% CI)** |  |
| **ICU LOS, days (IQR)** | -0.6 (-3.54 to 2.28) | 0.2 (-2.28 to 2.66) | -1.8 (-4.15 to 0.53) | -1.8 (-4.79 to 1.27) | 0.0 (-3.22 to 3.20) | -2.1 (-5.08 to 0.84) | -2.5 (-5.28 to 0.37) | -1.3 (-4.21 to 1.69) | -0.7 (-4.34 to 2.87) | -3.1 (-6.84 to 0.66) | 0.28 |
| **Hospital** LOS**, days (IQR)** | 1.8 (-4.21 to 7.75) | 0.3 (-5.77 to 6.29) | 0.4 (-5.67 to 6.38) | -4.5 (-10.43 to 1.37) | 3.1 (-2.73 to 8.99) | -1.6 (-7.89 to 4.67) | -3.3 (-8.97 to 2.34) | 1.4 (-4.74 to 7.48) | -3.4 (-9.76 to 2.94) | -2 (-8.96 to 4.87) | 0.32 |
| **Ventilator-free days @ D28, days (IQR)** | -0.2 (-3.14 to 2.66) | -1 (-3.93 to 1.93) | 0.8 (-2.04 to 3.54) | -2.5 (-5.28 to 0.36) | 3.5 (0.83 to 6.18) | 0.6 (-2.15 to 3.32) | 0.8 (-2.00 to 3.56) | 0.9 (-1.84 to 3.58) | -0.3 (-2.89 to 2.25) | 1.4 (-1.01 to 3.78) | 0.30 |
| **Vasoactive-free days @ D28, days IIQR)** | 0.0 (-2.90 to 2.81) | -0.7 (-3.60 to 2.29) | -0.4 (-3.25 to 2.52) | -2.2 (-4.99 to 0.67) | 3.9 (1.17 to 6.71) | -0.1 (-3.00 to 2.74) | 0.0 (-2.85 to 2.91) | 1.0 (-1.77 to 3.85) | -1.0 (-3.76 to 1.84) | 1.2 (-1.63 to 4.05) | 0.51 |
| **ICU-free days @ D28, days (IQR)** | 0.8 (-1.72 to 3.31) | -1.2 (-3.79 to 1.29) | -0.1 (-2.56 to 2.41) | -1.5 (-3.95 to 0.94) | 3.0 (0.64 to 5.35) | 0.8 (-1.69 to 3.19) | 0.6 (-1.83 to 3.08) | 1.1 (-1.27 to 3.52) | 0.5 (-1.8 to 2.78) | 1.6 (-0.61 to 3.72) | 0.20 |
| **Hospitalization- free days @ D90, days (IQR)** | -3.1 (-11.23 to 5.08) | -4.4 (-12.69 to 3.93) | -2.3 (-10.21 to 5.54) | -8.0 (-15.66 to -0.42) | 8.4 (0.55 to 16.20) | 2.6 (-4.99 to 10.17) | -0.6 (-8.39 to 7.24) | 0.5 (-6.91 to 7.86) | 2.3 (-5.27 to 9.91) | 4.1 (-2.89 to 11.13) | 0.05 |

**ACM=all-cause mortality, RR=risk ratio, MD=mean difference, LOS=length of stay, RRT=renal replacement therapy**

**Table S3. The effect of accelerated vs standard RRT strategy on outcomes based on baseline fluid balance ≤ or > 10% fluid overload**

| **Outcome** | **%Fluid overload ≤ 10** | **% Fluid overload > 10** | **p-value** |
| --- | --- | --- | --- |
|  | **RR (95% CI)** | **RR (95% CI)** |  |
| **90- day all-cause mortality** | 1.05 (0.93 to 1.18) | 0.83 (0.61 to 1.12) | 0.17 |
| **RRT dependence at 90 days** | 1.62 (1.12 to 2.38) | 1.90 (0.62 to 7.00) | 0.81 |
| **Death or RRT** | 1.09 (0.97 to 1.22) | 0.89 (0.66 to 1.19) | 0.20 |
| **ICU mortality** | 0.98 (0.85 to 1.13) | 0.96 (0.68 to 1.35) | 0.91 |
| **28-day mortality** | 1.05 (0.92 to 1.20) | 0.91 (0.65 to 1.28) | 0.44 |
| **Hospital mortality** | 1.05 (0.92 to 1.19) | 0.84 (0.62 to 1.15) | 0.22 |
|  | **MD (95% CI)** | **MD (95% CI)** |  |
| **ICU length of stay** | -1.2 (-2.23 to -0.25) | -2.5 (-5.73 to 0.69) | 0.37 |
| **Hospital length of stay** | -0.5 (-2.54 to 1.53) | -2.8 (-8.72 to 3.04) | 0.42 |
| **Ventilator-free days @ Day 28** | 0.3 (-0.64 to 1.26) | 1.2 (-0.94 to 3.29) | 0.50 |
| **Vasoactive-free days @ Day 28** | 0.1 (-0.85 to 1.09) | 0.9 (-1.57 to 3.28) | 0.59 |
| **ICU-free days @ Day 28** | 0.4 (-0.40 to 1.26) | 1.6 (-0.3 to 3.48) | 0.31 |
| **Hospitalization- free days @ Say 90** | -0.9 (-3.57 to 1.73) | 5.8 (-0.39 to 11.89) | 0.07 |
